# Effects of acute exposures to carbon dioxide on decision making and cognition in astronaut-like subjects

**DOI:** 10.1038/s41526-019-0071-6

**Published:** 2019-06-19

**Authors:** Robert R. Scully, Mathias Basner, Jad Nasrini, Chiu-wing Lam, Emanuel Hermosillo, Ruben C. Gur, Tyler Moore, David J. Alexander, Usha Satish, Valerie E. Ryder

**Affiliations:** 1 Biomedical Research and Environmental Sciences, KBRwyle, Houston, TX 77058 USA; 2 Biomedical Research and Environmental Sciences Division, Human Health and Performance Directorate, NASA Lyndon B. Johnson Space Center, Houston, TX 77058 USA; 3 Unit for Experimental Psychiatry, Division of Sleep and Chronobiology, Department of Psychiatry, University of Pennsylvania Perelman School of Medicine, Philadelphia, PA 19104 USA; 4 Brain Behavior Laboratory, Department of Psychiatry, Perelman School of Medicine at the University of Pennsylvania, Philadelphia, PA 19104 USA; 5 Space Medicine Operations Division, Human Health and Performance Directorate, NASA Lyndon B. Johnson Space Center, Houston, TX 77058 USA; 6 Department of Psychiatry and Behavioral Science, Upstate Medical University State University of New York, Syracuse, NY 13210 USA

**Keywords:** Risk factors, Psychology

## Abstract

Acute exposure to carbon dioxide (CO_2_) concentrations below those found on the International Space Station are reported to deteriorate complex decision-making. Effective decision-making is critical to human spaceflight, especially during an emergency response. Therefore, effects of acutely elevated CO_2_ on decision-making competency and various cognitive domains were assessed in astronaut-like subjects by the *Strategic Management Simulation* (SMS) and *Cognition* test batteries. The double-blind cross-over study included 22 participants at the Johnson Space Center randomly assigned to one of four groups. Each group was exposed to a different sequence of four concentrations of CO_2_ (600, 1200, 2500, 5000 ppm). Subjects performed *Cognition* before entering the chamber, 15 min and 2.5 h after entering the chamber, and 15 min after exiting the chamber. The SMS was administered 30 min after subjects entered the chamber. There were no clear dose–response patterns for performance on either SMS or *Cognition*. Performance on most SMS measures and aggregate speed, accuracy, and efficiency scores across *Cognition* tests were lower at 1200 ppm than at baseline (600 ppm); however, at higher CO_2_ concentrations performance was similar to or exceeded baseline for most measures. These outcomes, which conflict with those of other studies, likely indicate differing characteristics of the various subject populations and differences in the aggregation of unrecognized stressors, in addition to CO_2_, are responsible for disparate outcomes among studies. Studies with longer exposure durations are needed to verify that cognitive impairment does not develop over time in crew-like subjects.

## Introduction

Adverse effects of carbon dioxide (CO_2_) on cognitive processes have been reported,^1–3^ but the effects observed occurred at CO_2_ concentrations that were considerably higher than those deemed safe by regulatory agencies. However, studies^4,5^ using the *Strategic Management Simulation* (SMS) test to assess complex decision making demonstrated effects of CO_2_ on decision-making performance at or below 2500 ppm, a level that is half that of the permissible exposure limit for CO_2_ set by the Occupational Safety and Health Administration. The SMS detects cognitive deficits resulting from traumatic brain injury at decrement levels well below the threshold of sensitivity of traditional psychometric methods.^6,7^ Therefore, effects on cognitive functions observed with the SMS^4,5^ at surprisingly low levels of CO_2_ may be an outcome enabled by the greater sensitivity of the SMS to cognitive impairments.

On the other hand, the findings of effects of low concentrations of CO_2_ upon cognition are controversial and the literature is unsettled. No statistically significant effects on acute health symptoms or cognitive performance were seen during exposures of college students for 4.25 h to pure CO_2_ at 1000, 3000, or 5000 ppm.^8–10^ However significant decrements in cognitive performance were found when subjects were exposed to metabolically produced CO_2_ at 3000 and 5000 ppm.^8–10^ Zhang^8–10^ concluded that exposures to moderate concentrations of bioeffluents (BEs), but not CO_2_, will cause deleterious effects upon cognitive performance.

Disparity in outcomes that have assessed effects of CO_2_ on cognition are not limited to studies that have employed different methods of assessment. Recently, a study conducted at the Naval Submarine Medical Research Laboratory with 36 US submariners produced no significant differences in any SMS measures when results from CO_2_ exposures at 2500 and 15,000 ppm were compared to those at 600 ppm.^11^ The conflicting outcomes between that study^11^ and others^4,5^ that have used the SMS to assess effects of CO_2_ upon cognition recapitulates the conflict in outcomes obtained with traditional psychometric methods.^12^ This suggests that the reason for the disparate outcomes among studies is likely less related to differences among the cognitive tests used than to differences among other features of the studies.

It may be that characteristics of the subjects are important determinants in the outcome of the studies in which effects of CO_2_ upon complex decision making are assessed. The study of Satish^4^ involved a cohort of college-age students. In a different study, which included professional class employees, Allen^5^ found that performance on the SMS was adversely effected at concentrations as low as 950 ppm. On the other hand, a study performed by Rodeheffer^11^ using submariners of the US Navy, who are highly motivated and accomplished and who were admitted to their chosen profession after being screened by highly stringent processes that select applicants for their ability to maintain very high levels of performance while operating under duress in an extremely hostile environment, found no performance decrement on the SMS when the submariners were subjected to 2500 or 15,000 ppm CO_2_. It has been well established that different experience levels and age have an effect on the choice of decision making paradigm.^13–17^ Given the disparity in outcomes among the various studies however, there is no basis for predicting how CO_2_ would affect cognitive processes of astronauts.

Because it is not unusual for CO_2_ levels aboard the International Space Station (ISS)^18^ to exceed levels at which cognitive effects of CO_2_ were observed by Satish,^4^ and because thresholds for some clinically significant effects of CO_2_ are considerably lower in space than they are on the ground,^18^ it was important to determine whether the cognitive functions associated with complex decision making of crew-like subjects are affected by acute exposures to CO_2_ at concentrations that are routinely encountered aboard the ISS. Therefore, to examine the significance of the effects of acute exposures to CO_2_ on cognition within the contexts of NASA’s needs for behavioral health management and toxicity assessment, we have used the SMS to determine if acute exposures to CO_2_, at or below operationally relevant concentrations, affects cognitive functions of astronaut-like subjects.

The Spaceflight Cognitive Assessment Tool for Windows (WinSCAT) has been used operationally on the ISS on all expeditions. It provides crew surgeons with a tool to assess an astronaut’s cognitive status. WinSCAT is scheduled to be taken monthly but may be taken whenever a crewmember desires a self-assessment.^19,20^ However, WinSCAT may suffer from a ceiling effect, which occurs when high-performing subjects achieve perfect scores with no measureable difference between subjects at the ceiling level. Therefore reduced performance variance near the ceiling levels will result in an unreliable estimate of population performance variability. Traditional psychometric tests may show effects of severe trauma but not be sufficiently sensitive to assess or predict changes in operational efficiency that could have impacts on crew health, or safety.^19,21^ Thus for several reasons, including small sample size, learning effects, and lack of sensitivity, “our knowledge about cognitive effects of spaceflight is superficial”.^22^

A cognitive test battery, called *Cognition*, has been designed specifically to avoid a ceiling effect when assessing spaceflight crews. The 10 tests included in the battery cover a range of cognitive domains relevant for successful spaceflight operations and have been mapped to underlying neural substrates by functional magnetic resonance imaging (fMRI).^23,24^ Therefore, this tool provides bridges between cognitive models, neuroscience, and behavior, and is likely more sensitive in astronauts than tools that have been designed for a standard clinical population.

Spaceflight crews have often reported symptoms, such as problems concentrating, headaches, and on some occasions, dissatisfaction with their cognitive performance.^21^ The potential causes for performance decrements during space missions are many (e.g., CO_2_, fluid shifts, poor sleep, fatigue, stress, high workloads), but it is not possible to independently assess the effect of each in a space vehicle. Therefore a ground-based study, free of potential confounders that would be present during a space mission, was conducted in which effects of operationally relevant concentrations of CO_2_ on cognitive functions of astronaut-like subjects were assessed with *Cognition*^24^ and with the SMS.^4^ Because several components of *Cognition* assess cognitive functions that are important to adaptive decision-making, findings from *Cognition* also provide context for the interpretation of assessments of complex decision-making made with the SMS. This study provides a baseline terrestrial dataset for effects of CO_2_ on cognitive functions against which data collected with these tests during spaceflight may be compared.

## Results

Participants were randomly assigned to one of four groups. Six subjects were successfully recruited for all but the last of the four groups, which included four subjects. The 22 subjects included 14 men and 8 women. The average age for all participants was 38.8 (ranges 31–53 for men and 31–51 for women).

Subjects continuously wore wrist activity monitors (Actigraph wActiSleep-BT) for assessing sleep–wake patterns starting 1 week prior to the first exposure until after the last exposure. Actigraphy demonstrated very good compliance with the requirement to maintain their normal sleep durations (determined prior to the first exposure) during the course of the study. The average amount of night sleep during the week preceding exposure, and total sleep during the night preceding each of the exposures, did not differ significantly among the targeted CO_2_ concentrations. Although the amount of sleep during the night preceding each of the exposures did not differ significantly among the targeted CO_2_ concentrations, when investigated as a covariate, the amount of sleep by an individual preceding each exposure was found to be a significant covariate for the variable Initiative (*p* = 0.0332).

The data demonstrate that 600 ppm CO_2_ was maintained within ± 10% and the other three concentrations were maintained well within ± 5% of the targeted concentrations. The means and standard deviations for environmental variables at each of the targeted CO_2_ concentrations are given in Table 1. None of the environmental variables differed significantly among the targeted CO_2_ concentrations. Oxygen was maintained between 20.9% and 21.1%. Atmospheric pressure varied from a minimum of 755 mmHg to a maximum of 765 mmHg. Temperature and relative humidity of the subject-occupied area of the chamber were maintained in the ranges 67–72°F and 58–70%, respectively. With respect to noise levels in chamber, the total number of instances per hour in which the maximum level with A-weighted frequency response and slow time constant (*L*_AS,max_) exceeded 70 dB on any of three sound dosimeters over the course of the exposures ranged from 3 to 6.5, and the average level of *L*_AS,max_ in excess of 70 dB ranged from 71.5 to 74 dB among the targeted CO_2_ concentrations.

**Table 1:** Environmental parameters

CO_2_ target (ppm)	600	1200	2500	5000
O_2_ (%)	21.1 (0.02)	21 (0.01)	21 (0.01)	21 (0.03)
Press (mmHg)	760.1 (1.22)	759.4 (3.81)	760.4 (3.28)	760.8 (1.5)
Temp (°F)	70.1 (1.07)	68.9 (1.09)	69.9 (1.91)	70 (0.91)
Rel Hum (%)	62.1 (2.5)	66.1 (0.51)	64.7 (3.54)	62.3 (2.46)
Noise (# > 70/h)	5.5 (8.7)	4.4 (5.1)	4.7 (8.0)	3 (6.4)
Noise (ave > 70 dB)	71.5 (0.2)	74.2 (4.3)	72.1 (2.1)	72 (2.0)

Means and (standard deviations) of environmental parameters measured in the chamber during exposures to the various targeted CO_2_ concentrations (O_2_—oxygen; Press (mmHg)—pressure millimeters mercury; Temp (°F)—temperature Fahrenheit; Rel Hum—relative humidity; Noise (#>70/h – number of incidence per hour in which the highest sound pressure level recorded during a measurement interval of minute equaled or exceeded 70 dB(A) on any of the three sound dosimeters in the exposure chamber)

**Fig. 1:**
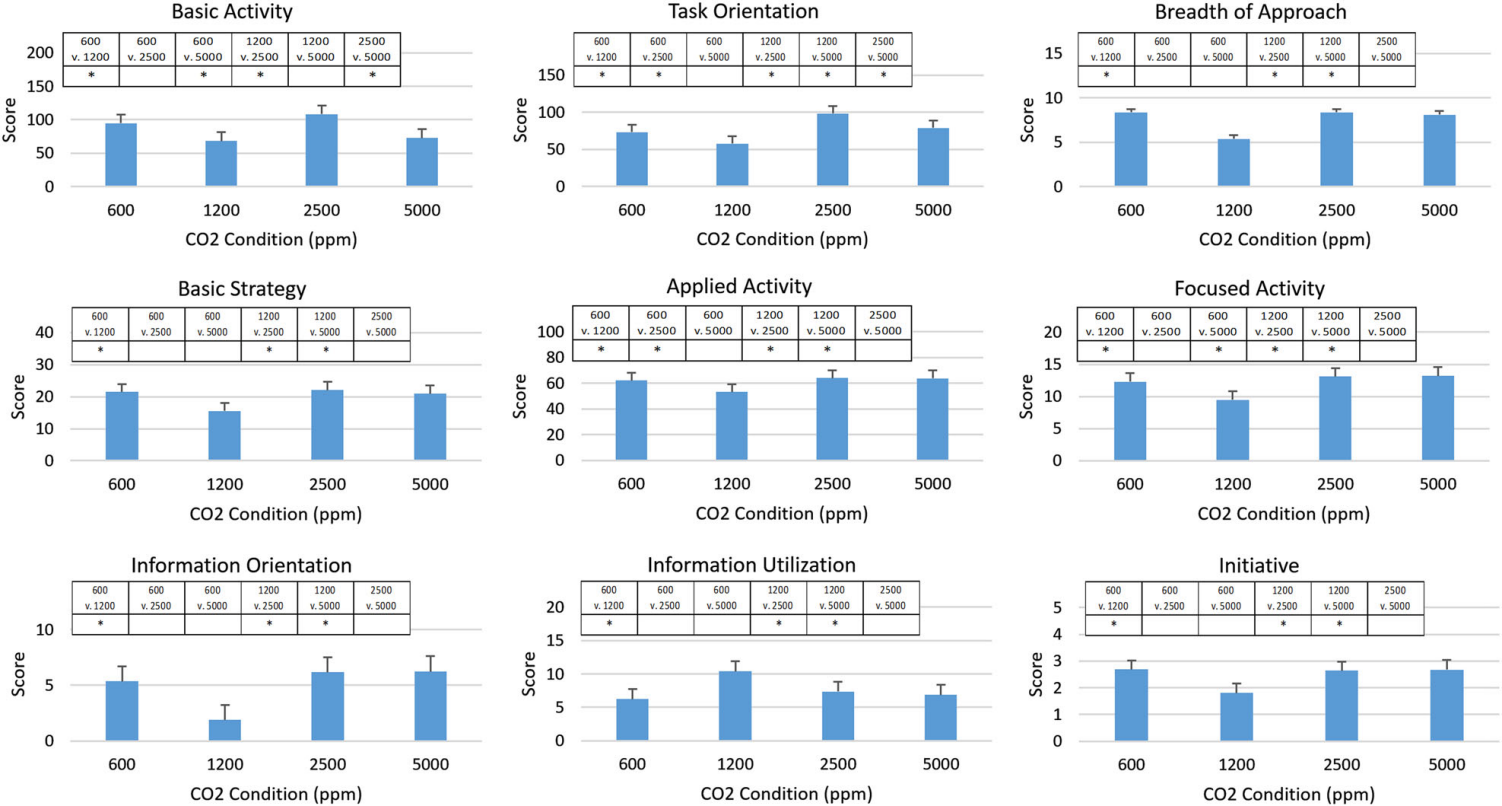
Means ± 95% confidence intervals of SMS measures at each targeted concentration of CO_2_. The raw scores assigned for each measure are linearly related to performance, with a higher score indicating better performance. Values are based on the relationship to established independent standards of performance among thousands of previous SMS participants.^4^ Measures for Initiative are the log-transformed values. *The threshold for significance used for post hoc comparisons by pairwise contrasts of adjusted predictions was *p* < 0.008, which was derived by dividing 0.05 by 6, the number of post hoc pairwise comparisons made

Estimated means of each of the SMS measures at each of the targeted concentrations of CO_2_ are shown in Fig. 1. All measures of complex decision-making changed significantly from their baseline values at 600 ppm when CO_2_ was increased to 1200 ppm (Fig. 1). For eight of the nine measures, scores decreased; however, for Information Utilization, the score increased at 1200 ppm. At 2500 ppm, only Task Orientation and Applied Activity scores were significantly different from baseline measures, and both measures exceeded their baseline values. At the highest concentration of 5000 ppm, again only two of the measures differed significantly from baseline. At this concentration, Focused Activity Level exceeded the baseline value, and Basic Activity was less than baseline.

**Fig. 2:**
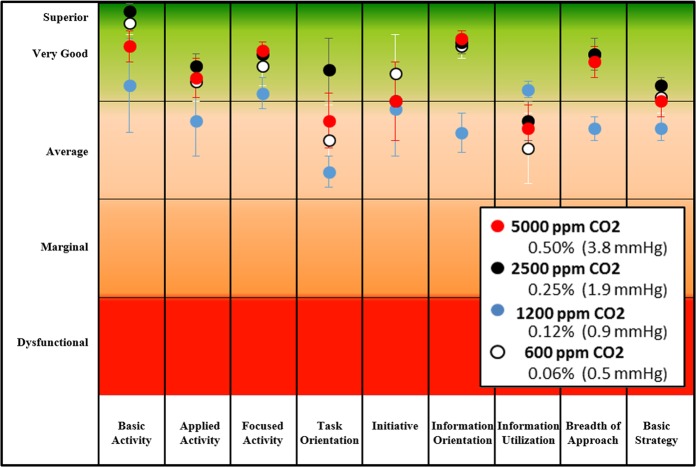
Mean ± 95% confidence intervals of percentile ranks for SMS measures at targeted concentrations of CO_2_. Decision-making performance scores were converted to percentile ranks by indexing against scores of performance measured in more than 20,000 subjects ages 16–83 years who were chosen to represent the working population of the US.^4^ The baseline is composed of responses by a variety of members of this population, including students, professionals, homemakers, and laborers

Raw scores that have been normalized to the percentile ranks are illustrated in Fig. 2. In contrast to a prior report,^4^ percentile ranks on all measures were always average or higher at all concentrations of CO_2_ targeted. Average percentile ranks were most often observed when subjects were exposed to 1200 ppm CO_2_, and better than average percentile ranks were the norm at the other concentrations tested.

**Fig. 3:**
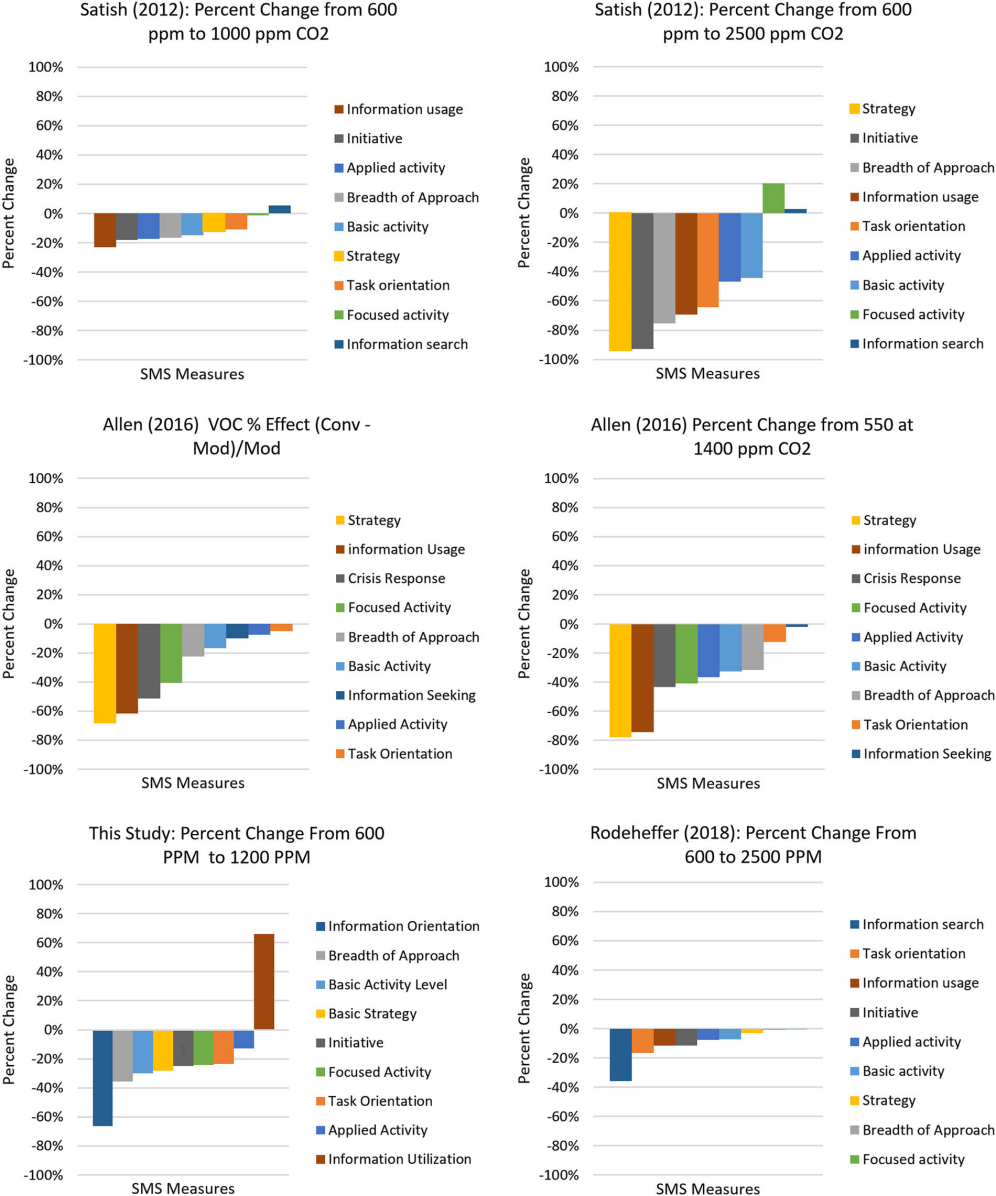
Percent change of SMS scores from baseline at elevated concentrations of indoor pollutants determined in several studies. When viewed as a percentage change from the baseline, the SMS measures that were most adversely affected differed among the studies but similarities in the set of most affected measures were greatest between the reports of Satish^4^ and Allen.^5^ In the Study of Allen^5^ most affected measures were the same for CO_2_ and VOCs. VOCs—volatile organic compounds

The effect on most SMS measures, as CO_2_ was increased from baseline to 1200 ppm, was a decrease in performance that was comparable to those observed in other studies.^4,5^ When viewed as a percentage change from the baseline (Fig. 3), the SMS measures that were most adversely affected differed among the studies but pairs of studies were similar to each other. Similarities in the set of most affected measures were greater when the study of Satish^4^ was compared to that of Allen^5^ and when this study was compared to that of Rodeheffer.^11^

**Fig. 4:**
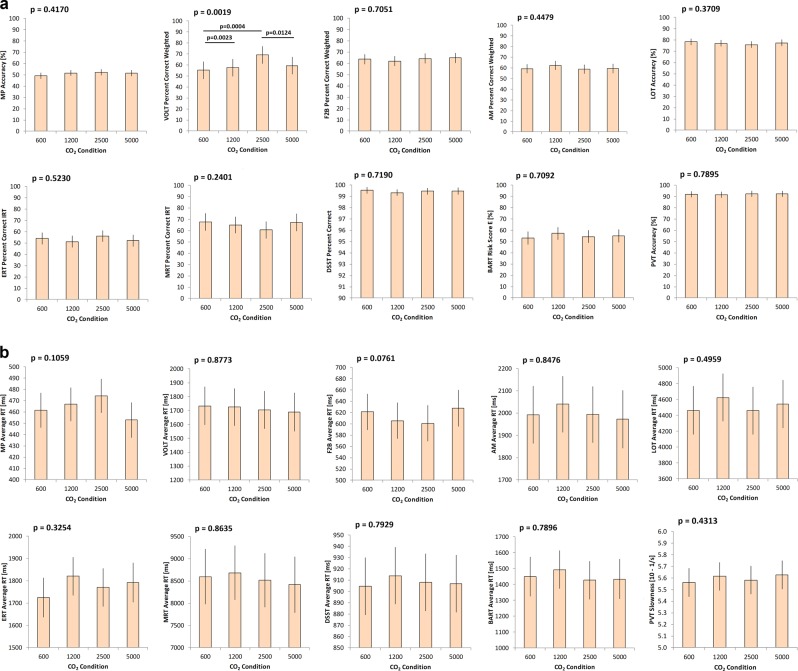
Mean ± 95% confidence intervals of accuracy (**a**) and speed (**b**) for the 10 cognition measures by group at each of the targeted CO_2_ concentrations (600, 1200, 2500, 5000 ppm). *p*-Values refer to Type-III fixed effects of variance (with *p* < 0.05 indicative for at least one concentration differing from the overall mean)

Raw scores for all *Cognition* tests were examined for outliers by multiple methods. Removal of data points flagged by the majority of methods as potential outliers produced no effect on outcomes, and therefore the analyses were conducted using the complete data set. Estimated means for accuracy and for speed for all *Cognition* measures, at each of the CO_2_ concentrations targeted, are shown in Fig. 4.

The *p*-values for summary statistics of *Cognition* results are provided in Table 2. Mixed models were used to estimate group least-square means and their differences, and to determine whether the difference was significantly different from 0 (LSMEANS statement in SAS). Only one of the 10 measures showed a statistically significant (*p* = 0.0019) difference from baseline (600 ppm). This was an improved score (Percentage Correct [PC]) on the Visual Object Learning Task (VOLT) at 2500 ppm. This difference remained significant at *p* < 0.05 after correcting for multiple testing with the false discovery rate method (*N* = 20 tests).^25^ Digital Symbol Substitution Task (DSST) and Psychomotor Vigilance Test (PVT) accuracy outcomes were transformed to binary outcomes (1 indicating 100% correct on the DSST and > 90% of non-lapse and non-false start responses on the PVT) and non-linear mixed effect models equivalent to model 1, described in Methods, were run. Likelihood ratio tests based on the full model and a model with CO_2_ condition removed indicated a significant effect of CO_2_ condition for the DSST (*p* = 0.0260). Regression model contrasts indicated that subjects were more likely to achieve 100% accuracy in the 2500 ppm condition relative to 5000 ppm (*p* = 0.0078). The estimated probabilities for 100% accuracy on the DSST were 72.3%, 72.8%, 80.9%, and 56.6% for 600, 1200, 2400, and 5000 ppm, respectively (estimates for test 1, session 1, and average pre-exposure probability of 61.4%). For the PVT, the probability of achieving an accuracy score of >90% decreased in a dose–response like fashion from 79.5%, 74.7%, 73.4%, to 64.0% for 600, 1200, 2400, and 5000 ppm, respectively (estimates for test 1, session 1, and average pre-exposure probability of 72.7%). However, there was no significant main effect of CO_2_ condition for the PVT (*p* = 0.4114).

**Table 2:** Cognition summary statistics

Variable	Main effects			Interaction	Contrasts						Main effects
	CO_2_ cond.	Exp. duration	Session #	CO_2_ cond.* duration	600 vs. 1200	600 vs. 2500	600 vs. 5000	1200 vs. 2500	1200 vs. 5000	2500 vs. 5000	After-effect
MP speed	0.1059	0.8086	0.4984	0.8637	0.5567	0.1571	0.3223	0.3828	0.1121	**0.0161**	0.3860
MP accuracy	0.450	0.3029	0.7805	0.5303	0.2096	0.1111	0.2722	0.7202	0.8946	0.6334	0.8427
VOLT speed	0.8773	**0.0191**	**0.0202**	0.6905	0.8907	0.6356	0.4601	0.7269	0.5389	0.7888	0.7223
VOLT accuracy	**0.0019**	0.1551	0.7220	0.9728	0.5420	**0.0004**	0.2916	**0.0023**	0.6382	**0.0124**	0.1537
F2B peed	0.0761	**0.0100**	0.8652	0.3641	0.588	0.0809	0.5948	0.6819	0.0621	**0.0239**	0.6651
F2B accuracy	0.7051	**0.0035**	**0.0245**	0.6867	0.5142	0.8185	0.6462	0.3728	0.2674	0.8145	0.3310
AM speed	0.8476	0.6425	0.1203	0.2936	0.5526	0.9883	0.7987	0.5573	0.3875	0.7861	0.3250
AM accuracy	0.4479	**0.0024**	0.3165	0.3488	0.2018	0.8830	0.951	0.1472	0.2443	0.8022	0.4071
LOT speed	0.4959	0.2458	0.454	0.5597	0.1992	0.9675	0.5468	0.1880	0.5097	0.5190	0.3511
LOT accuracy	0.3709	0.9494	0.5814	0.1183	0.3337	0.0835	0.5357	0.4245	0.7289	0.2638	0.3020
ERT speed	0.3254	**0.0034**	0.4444	0.5663	0.075	0.3868	0.2123	0.3409	0.5997	0.6876	0.3248
ERT accuracy	0.5230	0.7460	0.3973	0.3918	0.4160	0.5938	0.5714	0.505	0.8023	0.2702	0.2090
MRT speed	0.8635	**0.0000**	**0.0024**	0.4000	0.7883	0.8035	0.5851	0.5966	0.4125	0.7590	0.5603
MRT accuracy	0.2401	0.4792	0.8196	0.4449	0.4627	0.0711	0.9064	0.2500	0.5390	0.0853	0.0518
DSST speed	0.7929	0.8296	0.2421	0.7561	0.3301	0.7223	0.8086	0.5496	0.4719	0.9106	0.9424
DSST accuracy	0.7190	0.7902	0.3459	0.1386	0.2667	0.7124	0.7473	0.4616	0.4476	0.9712	0.4335
BART speed	0.7896	**0.0349**	0.1589	0.2956	0.5479	0.7550	0.8380	0.3490	0.4207	0.9188	0.6845
BART risk taking	0.6963	**0.0023**	**0.0122**	0.4598	0.2788	0.7030	0.3900	0.4739	0.8381	0.6229	0.6851
PVT speed	0.4313	0.0998	0.8280	0.1125	0.2239	0.6507	0.1453	0.4318	0.7940	0.3020	0.8271
PVT accuracy	0.7895	0.6324	0.1012	0.9718	0.8397	0.5903	0.4988	0.4540	0.3810	0.8780	0.2323

Summary statistics *p*-values (not adjusted for multiple testing) for effects of CO_2_ concentration, time in chamber, and session number, the interaction between CO_2_ concentration and exposure duration, contrasts between CO_2_ concentrations, and recovery post-exposure (After-Effect). The statistically significant (*p* = 0.0019) improved score (Percentage Correct [PC]) from baseline (600 ppm) on the Visual Object Learning Task (VOLT) at 2500 remained significant at *p* < 0.05 after correcting for multiple testing with the false discovery rate method.^15^ For direction of effects, see Fig. 4

Bold values indicate statistically significant *p*-values

The *Cognition* battery was administered early and late during the exposure period. Expected practice effects were noted for 5 of the 10 *Cognition* speed outcomes (Average Response Time [ART]) and accuracy on the Fractal 2 Back test (F2B) and Emotion Recognition Task (ERT) (Table 2), but no significant interaction between CO_2_ concentration and exposure duration could be found for any of the *Cognition* outcomes (all *p* > 0.05). Finally, *Cognition* performance post-exposure did not differ significantly between CO_2_ concentrations (adjusting for pre-exposure performance, all *p* > 0.05).

**Fig. 5:**
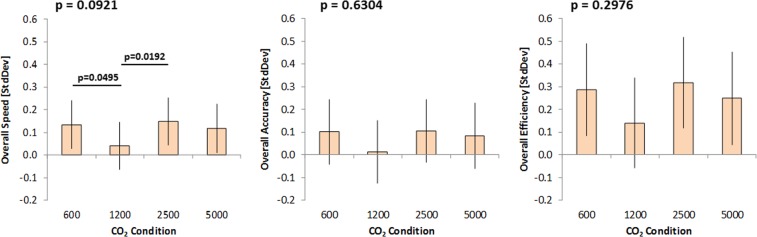
Evaluation of standardized scores of speed, accuracy, and efficiency across tests (higher scores reflect better performance). The *p*-values for significant differences in overall speed across tests achieved at different CO_2_ concentration are given on the graphs for Overall Speed. Error bars indicate the 95% confidence intervals

In addition to analyzing results of performance on the individual tests, analysis of aggregated standardized scores across tests was also performed. These data are summarized in Fig. 5. No significant main effect on speed (*p* = 0.0921), accuracy (*p* = 0.6304), or efficiency (*p* = 0.2976) were found, but response times, accuracy, and efficiency were lowest during exposures to 1200 ppm. While the overall effects were not statistically significant, they do indicate a trend for reduced accuracy, speed, and efficiency at 1200 ppm. However, performance across tests did not differ between baseline (600 ppm) and the higher concentrations.

The number of subjects that we assessed was based upon a power analysis of data from the study of Satish.^4^ Table 3 shows that the average coefficient of variation (CV) in our study at 2500 ppm (0.35) and 5000 ppm (0.34) was less than the CV at 1200 ppm (0.52) and also less than that of Satish^4^ at 2500 ppm (0.49) at which concentration effects on the SMS were pronounced. The CVs in the study of Rodeheffer,^11^ which reported no effects of CO_2_ upon performance on the SMS, ranged between 0.47 and 0.53. Therefore, the comparisons of CV among the studies indicates the absence of significant effects at our two higher concentrations was not a consequence of a greater variability, and hence less power to detect significant differences, at those concentrations.

**Table 3:** Coefficients of variation of measures of the SMS from several studies in which performance was assessed during exposures to CO_2_

Comparisons of means, standard deviations, and coefficients of variation among studies									
	Satish^4^			Rodeheffer^11^			This study		
	Mean	SD	CV	Mean	SD	CV	Mean	SD	CV
600 ppm									
Basic activity	69.59	7.04	0.10	89.92	31.62	0.35	94.8	32.2	0.34
Applied activity	117.86	39.28	0.33	54.58	24.24	0.44	62.1	14.7	0.24
Focused activity	16.27	3.2	0.20	12.33	4.48	0.36	12.3	3.2	0.26
Task orientation	140.82	28.66	0.20	90.33	35.44	0.39	73.2	17.3	0.24
Initiative	20.09	6.96	0.35	13.92	7.19	0.52	19.4	23.0	1.19
Information search	20.36	3.06	0.15	9.08	9.22	1.02	5.4	3.4	0.64
Information usage	10.32	3.21	0.31	8.58	5.05	0.59	6.2	3.5	0.56
Breadth of approach	9.36	1.36	0.15	7.83	1.47	0.19	8.4	0.7	0.09
Strategy	27.23	5.48	0.20	16.58	11.02	0.66	21.5	6.9	0.32
* Means*	47.99	10.92	**0.22**	33.68	14.41	**0.50**	33.69	11.67	**0.43**
1000 & 1200 ppm									
Basic activity	59.23	7.12	0.12				66.3	21.1	0.32
Applied activity	97.55	35.51	0.36				54.1	13.7	0.25
Focused activity	16.09	3.7	0.23				9.3	2.7	0.29
Task orientation	125.41	28.62	0.23				56.0	14.1	0.25
Initiative	16.45	6.7	0.41				14.6	23.0	1.58
Information search	21.5	3.2	0.15				1.8	1.9	1.04
Information usage	7.95	2.24	0.28				10.3	3.9	0.38
Breadth of approach	7.82	1.56	0.20				5.4	1.3	0.24
Strategy	23.95	5.65	0.24				15.4	5.3	0.34
* Means*	41.77	10.48	**0.25**				25.91	9.65	**0.52**
2500 ppm									
Basic activity	38.77	7.57	0.20	83.42	28.28	0.34	108.5	41.8	0.39
Applied activity	62.68	31.86	0.51	50.33	30.43	0.60	64.1	15.1	0.24
Focused activity	19.55	3.4	0.17	12.25	4.14	0.34	13.1	3.7	0.28
Task orientation	50.45	31.66	0.63	75.33	31.84	0.42	98.5	33.5	0.34
Initiative	1.41	1.26	0.89	12.33	8.28	0.67	15.4	7.5	0.49
Information search	20.91	3.08	0.15	5.83	6.02	1.03	6.2	3.9	0.64
Information usage	3.18	1.71	0.54	7.58	3.87	0.51	7.4	3.3	0.45
Breadth of approach	2.32	1.17	0.50	7.75	1.06	0.14	8.4	0.7	0.09
Strategy	1.68	1.32	0.79	16.08	12.13	0.75	22.1	6.0	0.27
*Means*	22.33	9.23	**0.49**	30.10	14.01	**0.53**	38.16	12.83	**0.35**
* 5000* *ppm*									
Basic activity							73.4	25.8	0.35
Applied activity							63.0	15.2	0.24
Focused activity							13.3	3.2	0.24
Task orientation							77.7	24.4	0.31
Initiative							15.9	7.6	0.48
Information search							6.3	3.4	0.54
Information usage							6.9	3.5	0.51
Breadth of approach							8.1	0.9	0.11
Strategy							20.8	5.9	0.28
* Means*							31.69	9.97	**0.34**
15,000 ppm									
Basic activity				89.58	21.47	0.24			
Applied activity				51.58	18.2	0.35			
Focused activity				11.5	3	0.26			
Task orientation				88.5	28.86	0.33			
Initiative				17.58	12.52	0.71			
Information search				8.92	7.46	0.84			
Information usage				8.58	5.43	0.63			
Breadth of approach				7.83	1.03	0.13			
Strategy				16	11.22	0.70			
* Means*				33.34	12.13	**0.47**			

Comparisons of CV among the studies indicates the absence of significant effects at our two higher concentrations was likely not due to greater variability, and hence less power to detect significant differences, at those concentrations

Bold values indicate means of the coefficients of variation among the SMS measures for each concentration used in the studies

None of the subjectively assessed outcomes differed significantly between CO_2_ exposure concentrations (*p* > 0.05). The estimated means of all outcomes were in the bottom half or third of the scale.

## Discussion

A principal aim of this study was to determine if the adverse effect of low concentrations of CO_2_ on the decision-making abilities of predominantly young college-age adults^4^ could be replicated in older astronaut-like individuals. Clearly, the dose-dependent, monotonic, reciprocal relationship between CO_2_ concentration and performance on the SMS that was demonstrated in earlier studies^4,5^ was not replicated in this study, which included concentrations within the ranges used in those earlier studies (Fig. 1 and 2). Interestingly, the response from baseline to 1200 ppm, for most measures, exhibited a decrease in performance that was comparable to those observed in other studies (Fig. 3). However, this trend did not hold in this study population at higher concentrations.

Our findings at 2500 and 5000 ppm diverge from those anticipated by the findings of earlier studies that demonstrated substantial effects of CO_2_ upon performance on the SMS at lower concentrations^4,5^ but the absence of an effect at 2500 ppm replicates the finding of Rodeheffer^11^ at that concentration. On the other hand we detect effects at 1200 ppm, as have other studies.^4,5^ Therefore, our findings, in part, both comport with and diverge from the finding of others.^4,5,11^ Several factors, discussed below, may contribute to our unusual and unexpected findings.

One potential variation among the studies that could affect differences in performance is the amount of sleep obtained by subjects preceding their exposures to CO_2_. Although the amount of sleep during the night preceding each of the exposures did not differ significantly among the targeted CO_2_ concentrations, the amount of sleep by an individual was a significant covariate for the variable Initiative (*p* = 0.0332). The sleep status of the subjects was not reported in the studies of Satish.^4^ In a study^26^ in which the SMS was utilized it was observed that an improvement of 25% in sleep score was associated with a 2.8% increase in cognitive function scores. If decrements follow a reciprocal relationship to that shown for improvements then, because our subjects averaged only 6.3 h of sleep during the nights preceding their exposures (the nightly average of the general population is 6.8 h^27^), the difference between the large percent decrease in cognitive scores seen in the study of Satish,^4^ and the absence of similar effects in this study at the same concentrations of CO_2_, could be expected to be attributable to differences in the sleep status of the subjects of the two studies only if sleep scores among Satish’s^4^ subjects were well below those of this study.

Differences in characteristics of various subject populations may account for diverging outcomes in studies assessing effects of CO_2_ upon decision-making. It may be that astronaut-like operations personnel and submariners, who are high-level performers, are more likely to have heightened situational awareness because of their stringent training. Therefore, these groups may develop faster adaptive patterns of responses and be more perceptive of their cognitive decline, and therefore may compensate more efficiently for self-perceived drops in performance than subjects drawn from the general population. Such distinctions could explain the differences in outcomes between college students^4^ and submariners^11^ to elevated CO_2_, but the decrements in performances of astronaut-like subjects that occurred when they were exposed to 1200 ppm CO_2_ are inconsistent with this account.

There is abundant evidence that the default decision-making paradigms of young and/or novice individuals differ from those of older and/or experienced individuals.^13–17^ The former most often make use of expected utility or compensatory decision-making paradigms and the latter are more likely to employ heuristics or noncompensatory mechanisms.^13–17^ If the decision-making paradigms were different among different subject populations and the SMS provides a more sensitive measure of one paradigm than the other, such circumstances could produce the disparities in outcomes that have occurred among studies that have utilized the SMS to assess the effect of CO_2_ upon complex decision making.^4,5,11^ Populations that are using similar decision-making paradigms may be more likely to share similarities in the subset of SMS measures most affected by CO_2_ than those that are using different decision-making paradigms. Fig. 3 illustrates greater similarity in most affected measures between the studies of Satish^4^ and Allen^5^ and between our study and that of Rodeheffer^11^ when the effect is measured as a percentage deviation from baseline values. A post hoc analysis of variance with the data from Table 3 showed that the means of individual measures of the SMS are most often significantly different between the study of Satish^4^ and that of Rodeheffer^11^ and between Satish’s study^4^ and our study, whereas there were few measures with a significant difference between our study and that of Rodeheffer^11^ (Table 4). The subjects of Satish’s study^4^ were predominately college students whereas subjects of the studies of Rodeheffer^11^ (US submariners) and this study (astronaut-like subjects) were older and principally from operations-oriented disciplines. The performance scores reported by Allen’s study,^5^ which involved professional-grade employees (architects, designers, programmers, engineers, creative marketing professionals, managers), were normalized to a unique experimental condition and so could not be directly compared to those of other studies. Our subjects exhibited performance decrements at 1200 ppm comparable in magnitude to those observed in Satish^4^ and Allen^5^ at similar concentrations. This finding indicates that the SMS is also sensitive to CO_2_-induced decrements in the decision-making paradigm that may be shared by astronaut-like subjects and submariners, which likely differs from that of the subjects of the studies of Satish^4^ and Allen.^5^ Therefore, we conclude that it is unlikely that disparities in outcomes among the studies that have assessed effects of CO_2_ on complex decision making with the SMS are due to differences in the sensitivity of the SMS to different decision-making paradigms used by the various subject populations. The disparities are more likely due to differing characteristics of the various subject populations and differences in the aggregation of unrecognized stressors, in addition to CO_2_.

**Table 4:** Significance differences of measures of the SMS from several studies in which performance was assessed during exposures to CO_2_

CO_2_ concentrations and SMS measures	1. Satish^4^	2. Rodeheffer^11^	3. This study	Differences among studies			
				*p*-Values			
				ANOVA	Tukey HSD		
	Mean	Mean	Mean		1 vs. 2	1 vs. 3	2 vs. 3
600 ppm							
Basic activity	69.59	89.92	94.8	**0.0048**	0.0732	**0.0047**	0.8528
Applied activity	117.86	54.58	62.1	**0.0000**	**0.0000**	**0.0000**	0.7455
Focused activity	16.27	12.33	12.3	**0.0006**	**0.0078**	**0.0012**	0.8860
Task orientation	140.82	90.33	73.2	**0.0000**	**0.0000**	**0.0000**	0.1801
Initiative	20.09	13.92	19.4	0.5116			
Information search	20.36	9.08	5.4	**0.0000**	**0.0000**	**0.0000**	0.1188
Information usage	10.32	8.58	6.2	**0.0028**	0.4096	**0.0019**	0.1936
Breadth of approach	9.36	7.83	8.4	**0.0014**	**0.0018**	**0.0239**	0.3721
Strategy	27.23	16.58	21.5	**0.0007**	**0.0006**	**0.0368**	0.1689
* Means*	47.99	33.68	33.69				
1000 & 1200 ppm							
Basic activity	59.23		66.3	0.1439			
Applied activity	97.55		54.1	**0.0000**			
Focused activity	16.09		9.3	**0.0000**			
Task orientation	125.41		56.0	**0.0000**			
Initiative	16.45		14.6	0.7190			
Information search	21.5		1.8	**0.0000**			
Information usage	7.95		10.3	**0.0196**			
Breadth of approach	7.82		5.4	**0.0000**			
Strategy	23.95		15.4	**0.0000**			
* Means*	41.77		25.91				
2500 ppm							
Basic activity	38.77	83.42	108.5	**0.0000**	**0.0001**	**0.0000**	**0.0343**
Applied activity	62.68	50.33	64.1	0.3121			
Focused activity	19.55	12.25	13.1	**0.0000**	**0.0000**	**0.0000**	0.7970
Task orientation	50.45	75.33	98.5	**0.0000**	0.0920	**0.0000**	0.1245
Initiative	1.41	12.33	15.4	**0.0000**	**0.0000**	**0.0000**	0.3464
Information search	20.91	5.83	6.2	**0.0000**	**0.0000**	**0.0000**	0.9667
Information usage	3.18	7.58	7.4	**0.0000**	**0.0003**	**0.0000**	0.9840
Breadth of approach	2.32	7.75	8.4	**0.0000**	**0.0000**	**0.0002**	0.1668
Strategy	1.68	16.08	22.1	**0.0000**	**0.0000**	**0.0000**	**0.0418**
* Means*	22.33	30.10	38.16				

Comparisons of the means of individual measures among the studies demonstrates that the means of measures from the study of Satish,^4^ are most often different from both this study and that of Rodeheffer,^11^ whereas the means of measures of Rodeheffer^11^ and this study are for the most part not statistically significantly different. These finding are consistent with a hypothesis that decision-making paradigms of the subjects of Satish may differ from those of Rodeheffer and this study, which likely do not differ significantly from each other. Additional evidence of distinctions in decisional strategies among subjects of the various studies that have used the SMS to assess effects of CO_2_ upon complex decision making is provided in Fig. 3

Bold values indicate statistically significant *p-*values

Because the decrements in performance on the SMS observed when 1200 ppm CO_2_ was targeted were not observed at higher concentrations of CO_2_, the possibility that the effect observed could have arisen from circumstances that were unique to conditions during exposure at 1200 ppm was considered. Ventilation rates differed between the exposures at 600 ppm and those at the three higher concentrations of CO_2_. When 600 ppm was targeted, CO_2_ produced metabolically by the subjects was prevented from accumulating by continuous operation of a blower that brought outside air into the third level of the chamber at 4.5 m^3^/min. CO_2_, when required, was introduced via the heating, ventilation, and air conditioning (HVAC) system, which at this targeted concentration was operated continuously at 5.4 m^3^/min. With all other targeted CO_2_ concentrations, the fresh air blower was disengaged and the HVAC flow was operated continuously at 5.1 m^3^/min. Therefore, accumulation of volatile organic compounds (VOCs) and/or BEs emitted by the subjects would be expected to be lowest when 600 ppm CO_2_ was targeted and higher during exposures to the other concentrations of CO_2_ during which no outside air was brought into the exposure chamber. Because accumulation of VOCs or BEs have measurable effects on performance on the SMS,^5,26–29^ it would be expected that if these agents contributed to the depressed performance at 1200 ppm then their effects should also have been evident when the two higher concentrations of CO_2_ were targeted unless these effects were alleviated by the higher concentrations of CO_2_. Increased CO_2_ blood concentrations elicit a number of physiological responses triggered by a pH-induced stimulation of central and peripheral chemoreceptors, including increases in heart rate and minute ventilation, cerebral arterial vasodilation, and central nervous system (CNS) arousal.^30–33^ For these reasons, it is plausible that a slight to moderate increase in CO_2_ levels increases CNS arousal and cognitive performance. However, the possibility of mitigation of effects of BEs by the higher levels of CO_2_ seems disallowed by reports of adverse effects on performance on the SMS^4,5^ in subjects exposed to CO_2_ at lower concentrations and ventilation rates sufficiently high to effectively purge BEs^4,5^ (Table 5).

**Table 5:** Exposure parameters

Study	Target CO_2_ (ppm)	Expos (h)	Subj # total	No. @ expos^a^	Chamber vol (m^3^)	Air flow rate	Air flow (m^3^/h)	Air flow (L/s)	Air flow (L/s/p)	Air Δ/h (no.)
This study	600	3	22	6	229	High	591	166	27.7	2.6
	1200, 2500, 5000	3	22	6	229	Low	302	85	14.2	1.3
Satish^4^	600, 1000, 2500	2.5	22	4	51	Steady	360	100	25.0	7.1
Maula^29^	540	4	36	6	209	High	609	169	28.2	2.9
	2260	4	36	6	209	Low	50	14	2.3	0.2
Zhang^8^	500, 1000, 3000	4.25	25	6	30	High	720	200	33.3	24.0
Zhang^9^	500	4.25	25	6	30	High	720	200	33.3	24.0
	1000	4.25	25	6	30	Low	155	43	7.2	5.2
	3000	4.25	25	6	30	Low	38	11	1.8	1.3
Zhang^10^	500, 5000	2.5	10	6	30	High	720	200	33.3	24.0

Exposure parameters of recent studies that have examined the effects of low concentrations of CO_2_ upon cognitive functions

^a^In the studies of Zhang, the number exposed included 1 experimenter

Findings converse to those discussed above^4,5^ have been reported^8–10^ from studies in which moderate accumulations of metabolically produced CO_2_ and accompanying BEs, but not exposures to identical concentrations of pure CO_2_, caused decrements in cognitive performances.^8–10^ The finding by Zhang^8–10^ provide no support for the hypothesis that adverse effects of VOCs and BEs may be mitigated by CO_2_ at our higher concentrations, unless the comparable levels of CO_2_ in Zhang’s studies were accompanied by substantially greater levels of BEs than those in our study. The levels of BEs were not reported by Zhang but they could have been well in excess of the levels of BE accumulated in this study because our targeted concentrations of CO_2_ were attained in a chamber volume that exceeded that of Zhang by a factor >2.5 (Table 5) and, unlike Zhang,^8–10^ exogenous CO_2_ had to be added to achieve our high targeted concentrations.

Mitigation of CO_2_ effects due to VOCs and BEs at the higher concentrations in this study may be refuted by the observation of performance decrements among office workers in locations described as afflicted with sick building syndromes. In these locations, high levels of VOCs and BEs are accompanied by elevated levels of CO_2._ However, in these settings, the sources of VOCs are potentially much greater than those in exposure chambers of controlled studies, and other environmental factors may be influencing performance as well.^28^

Although it is possible that CO_2_ at higher concentrations mitigates effects of BEs and/or VOCs in this study, in view of the disparate outcomes among this study and the various studies that have assessed the effects of CO_2_ upon complex decision making^4,5,11^ or general cognitive performance,^8–10^ it seems most probable that differing characteristics of the various subject populations and differences in the aggregation of unrecognized stressors, in addition to CO_2_, were responsible for the varied, disparate, and conflicting outcomes among these studies.

A principal objective in utilizing *Cognition* was to investigate whether performance on this test battery, which was specifically designed for the high-performing astronaut population, is affected by short-term exposure to levels of CO_2_ routinely occurring on the ISS. This ground-based study avoided other environmental stressors typically encountered on the ISS that could have confounded the effects of CO_2_ on cognition (e.g., fatigue, stress, high workloads) and permitted a direct assessment of the effects of brief exposures to low concentrations of CO_2_ on cognitive functions assessed by *Cognition*. A significant CO_2_ main effect was only observed for accuracy on the VOLT and for the probability to achieve perfect accuracy on the DSST. However, there was no clearly discernable dose–response pattern for any of the individual measures of *Cognition*.

When the results obtained with all *Cognition* measures were taken in aggregate, a slight decrease in performance at 1200 ppm relative to 600 ppm was observed. Performance with higher, but still modest, CO_2_ concentrations (2500 and 5000 ppm) were similar to performance at baseline (600 ppm). With effect sizes <0.2, the differences between CO_2_ conditions were small. This “dose–response” of performance on *Cognition* to CO_2_ recapitulates the dose–response obtained with the SMS test, which was administered during the same exposure sessions. It seems likely that the factors that were responsible for the dose–response pattern seen with the SMS, identified in the earlier discussion of results of the SMS, also produced the similar pattern in the aggregated scores of *Cognition*. The convergence of results obtained with *Cognition* and with the SMS provides confidence in results that differ significantly from those anticipated by the findings of Satish.^4^

The effects of short-term exposure to CO_2_ concentrations of up to 5000 ppm on *Cognition* performance were small and with no dose–response function that would indicate decreasing performance levels with increasing CO_2_ levels. Past studies on the effects of elevated CO_2_ levels on cognitive performance investigated substantially higher CO_2_ concentrations, and only some studies found effects on cognitive performance.^12^ As noted earlier, it is plausible that a slight to moderate increase in CO_2_ levels increases CNS arousal and cognitive performance. Based on the paucity of literature, symptom reports related to increased levels of CO_2_, and the CNS arousing properties of CO_2_, both positive and negative associations between CO_2_ levels and cognitive performance were plausible outcomes of our study.

The current findings suggest that performance on *Cognition* is not relevantly affected if astronaut-surrogate subjects are exposed to CO_2_ concentrations of up to 5000 ppm for less than 3 h. On the other hand, it could be that none of the 10 *Cognition* tests was sensitive enough to detect subtle CO_2_-induced changes in cognitive performance, or that the 10 tests did not cover those cognitive domains that would be considerably affected by elevated CO_2_. This is unlikely, however, as *Cognition* covers a range of cognitive domains and has been shown to be sensitive to other stressors like sleep loss,^34,35^ recovery from anesthesia,^36^ and head-down tilt bed rest.^35^ It is thus more likely that any observed effects induced by short-term exposure to CO_2_ concentrations of up to 5000 ppm were simply too subtle to induce relevant changes in performance on the measures of *Cognition*.

Interestingly, a recently published study on the effects of 12° head-down tilt with and without elevated levels of CO_2_ also found the VOLT as the most sensitive test relative to 5000 ppm CO_2_ levels.^35^ Therefore, it could be that the medial temporal cortex and the hippocampus are especially sensitive to changes in CO_2_ concentration, with concomitant changes in memory performance.

SMS and *Cognition* test performances assessing a range of cognitive domains important for safe spaceflight operations suggest minor effects of an exposure for <3 h to CO_2_ concentrations of up to 5000 ppm in the investigated ground-based population. Both the SMS and *Cognition* demonstrated a slight performance decrease at 1200 ppm relative to 600 ppm. Our results are unique and comport with neither those of Satish^4^ or Rodeheffer,^11^ which conflict with each other in their conclusions regarding the effect of CO_2_ on complex decision-making as assessed by the SMS. It is possible that the effects we observed on both the SMS and *Cognition* may be due to accumulated VOCs and BEs, and the recovery of performance with higher but still modest CO_2_ concentrations may be related to the excitatory and vasodilatory properties of CO_2._ However, in view of the disparate outcomes among this study and the various studies that have assessed the effects of CO_2_ upon complex decision making^4,5,11^ or general cognitive performance,^8–10^ it seems most probable that differing characteristics of the various subject populations and differences in the aggregation of unrecognized stressors, in addition to CO_2_, were responsible for the varied conflicting outcomes among these studies. Environmental control and life support systems of spacecraft are required to avoid accumulation of VOCs and BEs. Additional studies of acute exposures, along with studies of longer exposure durations and studies that evaluate the effects of acute CO_2_ spikes on top of an elevated background, are needed to further evaluate potential adverse impacts of CO_2_ on decision-making and cognition during spaceflight operations.

## Methods

The study was reviewed and approved by the Institutional Review Board (IRB) of the Johnson Space Center (JSC). Written informed consent was obtained from the human participants who took part in the study. Twenty-two healthy, astronaut-like persons at the JSC were recruited by the Human Subject Test Facility at JSC to participate in this investigation. Volunteer subjects were selected according to inclusion criteria that are used in the selection of astronaut candidates (see Subject Criteria in Supplementary Method). Exclusion criteria (see Subject Criteria in Supplementary Method) were used to avoid potential risks to the subject or study.

A double-masked format was used in which both the subjects and the experimenters and data analysts were unaware of the CO_2_ concentrations used during any of the exposure sessions. Our experimental design involved four groups, each composed of 4–6 subjects who participated in repeated trials of the experiment under varying concentrations of CO_2_. Subjects participated in each of four different conditions: 600, 1200, 2500, and 5000 ppm CO_2_. Each group was exposed to one concentration of CO_2_ on 1 day in each of 4 consecutive weeks. We randomized groups to one of four different dose exposure sequences (dose orders: A, C, B, D; B, D, A, C; D, A, C, B; C, B, D, A).

**Fig. 6:**
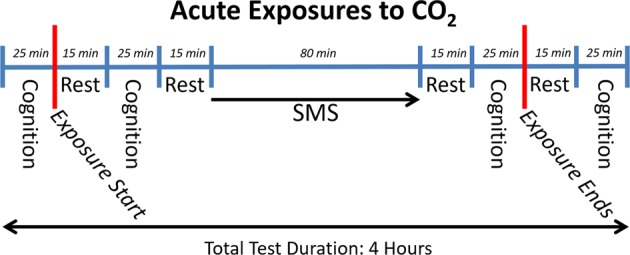
Sequence and durations of events on days of exposure. The sequences and duration of tests and intervening rest periods on days of exposure are indicated on the time line

Each group was exposed for ~3 h in the morning, on 1 day each week, for 4 consecutive weeks. Groups 1 and 2 completed their full sequence of exposures before exposure sessions were begun with groups 3 and 4. Each session included the steps and intervals illustrated in Fig. 6.

Significant work responsibilities prevented some subjects from attending all sessions. After permission of the IRB at the JSC was secured, additional sessions were scheduled with subjects who were willing to reschedule a missed session. As with the regularly scheduled sessions, only the chamber operators were cognizant of CO_2_ concentrations targeted in the rescheduled sessions. Two of these sessions targeted 1200 ppm for members of group 2, others targeted 600 and 2500 ppm for members of group 4, and 5000 ppm for a member of group 3. Sessions could not be rescheduled for three subjects, members of groups 1, 2, and 4. Two of the subjects missed sessions in which 5000 ppm was the targeted exposure concentration and the third missed a session in which 1200 ppm was targeted. The use of makeup sessions resulted in no unique exposure sequences. In all cases, subjects who did not complete the full complement of exposures had the best performances (aggregate score for all SMS measures) at 2500 ppm. The actual subject-groupings and exposure sequences are shown in Supplementary Table 1 that is available on-line.

Information on the quantity and quality of sleep of each subject was provided by data from actigraphy and sleep logs. Subjects were required to wear an actigraphy watch (Actigraph wActiSleep-BT) for 7 days before their first exposure and throughout their entire participation in the study.

Exposures were performed on the first floor of a human-rated three-story, 20-foot chamber (229 m^3^ total volume) at JSC. The facility was configured to support the safe evaluation of human subjects at elevated concentrations of CO_2_ for a period up to 4 h at sea level, with normal O_2_, and room temperature conditions. A Pressure Control System was modified (both mechanically and via firmware) to provide the introduction, monitoring, and control of CO_2_ for the chamber. The chamber has an adjustable HVAC system and a dedicated two-speed positive pressure blower. Both were used to maintain temperature and humidity, and CO_2_ in the desired ranges. To maintain CO_2_ at the lowest concentration targeted, a blower was used to prevent accumulation of CO_2_ produced metabolically by the subjects. This blower augmented ventilation provided by the HVAC (5.4 m^3^/min) by bringing outside air into the third level of the chamber at a rate of 4.5 m^3^/min. For all other targeted CO_2_ concentrations, the fresh air blower was disengaged, and the HVAC flow was decreased to 5.1 m^3^/min. Two high-resolution (0–5000 ppm) and two low-resolution (0–7000 ppm) sensors were located on the first level, and two low-resolution sensors were located on the unoccupied second and third levels. In addition to CO_2_, oxygen content, relative humidity, pressure, and temperature were monitored and recorded for the first level of the chamber. Three noise dosimeters were distributed in the exposure chamber. These dosimeters were accurate between 70 and 140 dB.

The primary outcomes for the study are the cognitive performance measures provided by the same SMS software that had been used to examine the effects of elevated levels of CO_2_ on aspects of cognitive decision-making by college students.^4^ Nine cognitive scores (each derived from multiple measurements built into a computer program that subjects interact with) were assessed under three different CO_2_ levels 600, 1000, and 2500 ppm. The factor scores resulting from the SMS software are continuously scaled and normally distributed, and appropriate for analysis by standard parametric statistical methods. From these data, we extracted the means, variability measures, and correlations among repeated measures necessary to derive power curves that associate the likelihood of detecting effects of similar magnitude among these three levels of CO_2_ on the nine cognitive factors. Power analysis indicated that a minimum *n* of 20–25, would be sufficient to exceed 80% power to detect differences between 1200 and 2500 ppm on all nine of these cognitive factors, and five of the nine factors in the 600 vs. 1200 ppm comparisons.

We used the SMS^4^ (Upstate Medical University, State University of New York) in our assessment of effects of each of four concentrations of CO_2_ on cognitive functions. SMS test simulations are broad, open-ended performance-based test scenarios that assess wider range of neural substrates than those that assess one or a small subset of executive functions. Therefore, a broader survey may provide a greater range within which to detect decrements.^4^ The SMS is unique in that it assesses the process of adaptive decision-making (planning, execution, and monitoring), whereas other psychometric tests typically assess individual or more limited sets of executive functions. Executive functions are high-level abilities that influence more basic functions, and include initiation, planning, sequencing, monitoring (attention), problem solving, working memory, divided attention, flexibility, and motor skills.^37^ Executive functions are important for adaptation and performance in real-life situations. In real world settings, options, priorities, and requirements are not always evident, outcomes depend on self-initiated actions and monitoring, and the effects of choices and actions may not be apparent. The SMS test simulations expose subjects to situations in which decisions must be made in conditions of volatility, uncertainty, complexity, and with delayed feedback.^38^ Decision-making competence is assessed in the SMS by *how* information is applied to make a decision. This is in contrast to assessments of decision-making that assess *what* was decided.

Prior to the first testing session, subjects were provided with a training session in which they were familiarized with the operation of the SMS during an abbreviated presentation of a scenario. Four scenarios were used during the study. Each of the four scenarios was used once with each group, the order of presentation of the scenarios was the same in each group and therefore the CO_2_ concentrations during which each scenario was presented differed among groups. The availability of multiple scenarios allowed retesting of subjects greatly reduces bias due to experience and learning effects, and intra-subject variability is low.^4^ Scenarios were presented to subjects via personal computer along with a variety of options to deal with the circumstances presented, including the option to do nothing. All subjects received the same quantity of information at fixed points in the simulated time, but actions could be taken and decisions made at any time during the simulation. Subjects, therefore, as in the real world, were not constrained to a particular action, plan, or strategy style. The SMS calculated raw scores based on the actions taken in response to incoming information, and information available earlier, and outcomes and their stated plans. More than 80 computer-gathered measures, which have been identified in earlier simulation studies as optimal predictors of success in complex decision making and subjected to multiple stepwise regression procedures to identify intercorrelations among simulation measures, are loaded on reliable and independent factors based on factor analytic varimax rotation of data collected from more than 20,000 subjects.^7,38–43^ The validated measures, which are derived from complexity theory, vary from assessments of simple competencies, such as speed or response and task orientation, to initiative, use of information, breadth of approach to problems, planning capacity, and strategy (Table 6). The measures have been validated by successfully predicting success among individuals engaged in positions exercising considerable complex perceptual and decision-making tasks.^7,38–43^ Decision-making performance scores were converted to percentile ranks by indexing against scores of performance measured in more than 20,000 subjects ages 16–83 who were chosen to represent the working population of the US.^4^ The baseline is composed of responses by a variety of members of this population, such as from students, professionals, homemakers, and laborers.

**Table 6:** Descriptions of measures of the SMS^4,39^

SMS measure	Description
Basic Activity Level	Overall competence to make decisions at all times
Task Orientation	Competence to make specific decisions that affect completion of current tasks.
Breadth of Approach	Competence to use multiple options and opportunities to achieve goals
Basic Strategy	Competence to make effective use of information and planning
Applied Activity Level	Competence to make decisions that are relevant to achievement of overall goals
Focused Activity Level	Capacity to remain attentive to current situations
Information Orientation	Competence to collect, as required, available information
Information Utilization	Capacity to use both provided and collected information toward attaining overall goals
Initiative	Development of new/creative activities

Raw scores for all measures at all points during all sessions were examined for outliers by inspection of scatter plots, box plots, and plots of Cooks’s distance, covariance ratios, robust regression residuals vs. robust distance, and examinations of Studentized residuals. Removal of data points flagged by at least three methods as potential outliers (11 of 850 data points), produced no effect on outcomes, and therefore the analyses were conducted using the complete data set. Statistical analysis software (Stata 14.1, College Station, TX; SAS 9.4, Carey, NC) was used for analyses, employing hypothesis-driven two-tailed alpha to reject the null hypothesis at 0.05. The main-effect variable examined, concentration of CO_2_, was treated as a categorical variable with values of 600, 1200, 2500, and 5000 ppm. Statistical assumptions were tested in concert with all techniques, and appropriate data transformations were used as needed to meet these assumptions. Values of the variable *Initiative* were transformed to their logarithms to meet criteria required for parametric analyses.

All of our primary outcome measures described above are continuously scaled, and all followed a normal distribution (or could be normalized) so that standard parametric statistical techniques were used. For these outcomes, we submitted the data to separate (per outcome) mixed-effects analyses that included both repeated-measures ANOVA (with SAS) and repeated-measures (subject) random intercept restricted maximum-likelihood method (Stata) to accommodate the repeated-measures experimental design. Our preliminary models included main effects and an interaction term for a variable in order to determine if the variable influenced performance outcomes. We independently assessed age, gender, session, and sleep durations preceding exposures as covariates. The amount of sleep by an individual preceding each exposure was found to be a significant covariate for the variable Initiative. Otherwise no effect for any factor was observed, so we reverted to a primary model comparing effects of the various concentrations of CO_2_ on each of the SMS factors. When significant differences were determined to exist among effects of concentrations, post hoc analyses among multiple pairs of concentrations were conducted using both Diffograms (mean—mean scatter plot) produced with Proc GLIMMIX (SAS) and pairwise contrasts of adjusted predictions (Stata) to determine which concentrations differed. The threshold for significance used was 0.008, which was derived by dividing 0.05 by 6, the number of post hoc pairwise comparisons made.

Before taking each *Cognition* test battery, subjects filled out a 10-item Likert-type (range 0–10) survey that asked, “How are you feeling now?”. The questions had the following anchors: Not sleepy at all–Very sleepy, Happy–Unhappy, No headache–Severe headache, Energetic–Physically exhausted, Mentally sharp–Mentally fatigued, Not stressed at all–Very stressed, Not confused at all–Very confused, No shortness of breath–Severe shortness of breath, No problems concentrating–Severe problems concentrating, Heart beating normally–Heart racing. The survey also asked subjects to identify items consumed, including food, drink, smoking, medications, and to indicate the quantities and times of consumption. The times of the start and ending of any strenuous activities was also requested.

We implemented a version of the *Cognition* battery of psychometric tests as described by Basner^23^ and by Moore.^44^ The tasks are “touch-based cognitive tasks” administered via an iPad. Data (metrics, metadata, and configuration data), as well as comments that can be entered by subjects, were recorded at the completion of each task. The component tasks of the *Cognition* battery, the cognitive domains involved, the primary brain areas recruited for each task, and the average duration for each task are shown in Table 7.

**Table 7:** Cognition Tasks: The table identifies the cognitive domain, brain areas primarily recruited in performing the task and the time required to administer the task^23^

Task name	Cognitive domain	Brain regions primarily recruited	Average admin time (min)
Motor Praxis Task (MPT)	Motor speed	Sensorimotor cortex	0.5
Visual Object Learning Task (VOLT)	Visual learning and spatial working memory	Medial temporal cortex—hippocampus	1.7
Fractal 2-Back (F2B)	Working memory	Dorsolateral prefrontal cortex, cingulate, hippocampus	1.9
Abstract Matching (AM)	Abstraction	Prefrontal cortex	2.4
Line Orientation Task (LOT)	Spatial orientation	Right temporo-parietal cortex, visual cortex	2.1
Digital Symbol Substitution Task (DSST)	Complex scanning and visual tracking	Temporal cortex, prefrontal cortex, motor cortex	1.6
Balloon Analog Risk Task (BART)	Risk decision making	Orbital frontal cortex, amygdala, hippocampus, Anterior cingulate cortex	2.3
Psychomotor Vigilance Test (PVT)	Vigilant attention	Prefrontal cortex, motor cortex, visual cortex	3.2
Matrix Reasoning (MR)^53^	Abstract reasoning	Frontal, parietal	4
Emotion Recognition Task (ERT)^54^	Emotion recognition	Temporo-limbic regions	1.8

The *Cognition* test battery consists of 10 brief neurocognitive tests (tasks) that cover a range of cognitive domains (Table 7). These include executive control, memory, attention, emotional processing, risk decision-making, abstraction, and sensorimotor speed. It was specifically designed for high-performing astronauts, and consists of 15 unique versions that allowed repeated administration of the battery with minimal re-use of the same stimuli. Importantly, brain regions involved in performing each of the *Cognition* tests have been established with fMRI, and the tests that are the basis for *Cognition* have been well validated in both healthy individuals (e.g., 60,000 soldiers in the Army STARRS project)^45^ and patient populations.^46^ *Cognition* was performed on a fourth-generation iPad in this study.

*Cognition* consists of the following 10 cognitive tests (for a detailed description of the battery see Basner^23^): The *Motor Praxis Task* is a measure of sensory-motor speed and taps the sensorimotor cortex.^47^ Participants had to mouse click on ever-shrinking blue boxes that appeared in varying locations on the screen. The *VOLT* is a measure of visual object learning and memory, and links to the medial temporal cortex and the hippocampus.^48^ Participants had to remember and later recognize ten 3D Euclidean shapes. The *Fractal-2-Back* is a measure of attention and working memory related to the dorsolateral prefrontal cortex, cingulate cortex, and hippocampus.^49^ Fractal images were projected at 1 Hz and participants were asked to press the spacebar whenever the fractal on the screen was the same as the fractal before the previous one (2 back). The *Abstract Matching Task* is a measure of abstraction and recruits prefrontal cortex.^50^ Participants were asked to pair a central target object with two objects on either the left or the right lower side of the screen. The *Line Orientation Task* is a measure of spatial orientation ability, based on Benton’s test, and activates the right temporo-parietal cortex and the visual cortex.^51^ In each trial, participants were asked to rotate a moveable blue line of variable length so that it is parallel to a fixed black line. The *Emotion Recognition Task* recruits the cingulate cortex, amygdala, hippocampus, and fusiform face area.^52^ Participants were shown a series of faces and asked to determine what emotion each face was showing: happy, sad, anger, fear, or no emotion. Difficulty was varied by emotion intensity. The *Matrix Reasoning Task* is a measure of abstract reasoning and consists of increasingly difficult pattern-matching tasks.^47,53,54^ It is analogous to Raven Progressive Matrices^55^ and recruits prefrontal, parietal, and temporal cortices.^54^ The *Digit Symbol Substitution Task* involves matching numbers to symbols and is a measure of complex scanning, visual tracking, and processing speed.^56–58^ It relates to temporal, prefrontal, and motor cortices. The *Balloon Analog Risk Task* is a measure of risk decision-making and recruits the orbital frontal cortex, amygdala, hippocampus, and anterior cingulate cortex.^59^ Participants bet by inflating 30 computerized balloons, with larger balloons offering greater but riskier rewards since no reward is given if the balloon “explodes”. The 3-min *Psychomotor Vigilance Test* measures vigilant attention by recording reaction times to visual stimuli that appeared at random inter-stimulus intervals.^60–62^ It relates to prefrontal, motor, and visual cortices. *Cognition* was administered before, during (early and late), and after each exposure session.

For each of the 10 *Cognition* tests, one key accuracy outcome and one key speed outcome were analyzed using a linear mixed-effects model with restricted maximum-likelihood estimation. Random-effects intercept terms per subject were used to accommodate the repeated-measures experimental design. For each outcome variable, we calculated four separate models:

1. Discrete CO_2_ effect model: Independent variables included CO_2_ condition (four levels), experimental session (four levels), time in CO_2_ (two levels), and pre-exposure performance (continuous variable).
2. CO_2_ effect by time in CO_2_ interaction model: As model 1, but including a CO_2_ condition/time in CO_2_ interaction term.
3. Recovery model: Independent variables included CO_2_ condition (four levels), experimental session (four levels), and pre-exposure performance (continuous variable).
4. Continuous CO_2_ effect model: Independent variables included CO_2_ exposure level (continuous), CO_2_ exposure level squared (continuous), and pre-exposure performance (continuous variable).

For models 1, 2, and 4, data were restricted to the measurements performed in the chamber. For model 3, data were restricted to the post-exposure measurement. Least-squares estimation was used to produce predicted average scores and confidence limits for each dose level by predicting the marginal means over a balanced population. Q–Q plots of model residuals were checked for normality. Only residuals for models with DSST percent correct and the PVT accuracy as outcomes did not follow a normal distribution. These outcomes were transformed to binary outcomes (100% accuracy was coded as 1, and 0 otherwise). We then ran non-linear mixed effect models for model 1 above. Four subjects were identified being potentially non-compliant on one test (*N* = 3 subjects) or two tests (*N* = 1 subject). In sensitivity analyses, analyses were repeated without these subjects. A total of 111 (or 3.0%) out of 3740 expected test bouts were missing due to absent subjects or subjects logging in with the wrong ID.

Speed and accuracy scores across tests were generated by first z-transforming each outcome based on the mean and standard deviation of the four pre-exposure tests calculated using the data of all subjects, and then averaging z-transformed scores across the 10 tests (speed scores were multiplied by −1 so that higher scores reflected faster speed). MPT, DSST, BART, and PVT were not included in the calculation for the accuracy score, as subjects were not asked to hit the center of the square (MPT), PVT, and DSST primarily address speed, and BART primarily addresses risk taking and not accuracy. For the ERT and MRT, we used weighted scores based on Item Response Theory analyses of individual stimuli. Efficiency scores were calculated by averaging speed and accuracy scores. Data from tests of non-compliant subjects were excluded from standardization and analysis (i.e., 0.6% of data excluded). All *Cognition* data were analyzed using SAS v9.4.

### Reporting Summary

Further information on experimental design is available in the Nature Research Reporting Summary linked to this article.

## Supplementary information

Reporting Summary

Supplementary Information

## Data Availability

Data from this study can be obtained through a “Data Request” in the NASA Life Science Data Archive (https://lsda.jsc.nasa.gov/Request/dataRequest). The study title “Effects of Acute Exposures to Carbon Dioxide upon Cognitive Function (Acute_CO2_Exposure)”, and the specific data requested, should be entered into the “Data Request Description”.

## Supplementary information

**Supplementary Information** accompanies the paper on the *npj Microgravity* website (10.1038/s41526-019-0071-6).

## References

[1] Manzey D, Lorenz B (1998). Joint NASA-ESA-DARA Study. Part three: effects of chronically elevated CO_2_ on mental performance during 26 days of confinement. Aviat. Space Environ. Med..

[2] Sayers JA, Smith REA, Holland RL, Keatinge WR (1987). Effects of carbon dioxide on mental performance. J. Appl. Physiol..

[3] Selkirk, A., Shykoff, B. & Briggs, J. *Cognitive Effects of Hypercapnia on Immersed Working Divers*. Report No. NEDU-TR-10-15 (Navy Experimental Diving Unit, Panama City, FL, 2010).

[4] Satish U (2012). Is CO_2_ an indoor pollutant? Direct effects of low-to-moderate CO_2_ concentrations on human decision-making performance. Environ. Health Perspect..

[5] Allen JG (2016). Associations of cognitive function scores with carbon dioxide, ventilation, and volatile organic compound exposures in office workers: a controlled exposure study of green and conventional office environments. Environ. Health Perspect..

[6] Streufert S (1993). Alcohol and complex functioning. J. Appl. Soc. Psychol..

[7] Streufert S, Pogash R, Piasecki M (1988). Simulation based assessment of managerial competence: reliability and validity. Pers. Psychol..

[8] Zhang X, Wargocki P, Lian Z (2017). Physiological responses during exposure to carbon dioxide and bioeffluents at levels typically occurring indoors. Indoor Air.

[9] Zhang X, Wargocki P, Lian Z, Thyregod C (2017). Effects of exposure to carbon dioxide and bioeffluents on perceived air quality, self‐assessed acute health symptoms and cognitive performance. Indoor Air.

[10] Zhang X, Wargocki P, Lian Z (2016). Human responses to carbon dioxide, a follow-up study at recommended exposure limits in non-industrial environments. Bldg. Environ..

[11] Rodeheffer CD, Chabal S, Clarke JM, Fothergill DM (2018). Acute exposure to low-to-moderate carbon dioxide levels and submariner decision making. Aerosp. Med. Hum. Perform..

[12] Stankovic, A., Alexander, D., Oman, C. M. & Schneiderman, S. *A Review of Cognitive and Behavioral Effects of Increased Carbon Dioxide Exposure in Humans*. NASA/TM-2016-219277 https://ston.jsc.nasa.gov/collections/TRS/_techrep/TM-2016-219277.pdf (2016).

[13] Gigerenzer, G., Hertwig, R. & Pachur, T. in *Heuristics: The Foundations of Adaptive Behavior—Introduction* (eds Gigerenzer, G., Hertwig, R. & Pachur, T.) (Oxford University Press, Oxford, England, UK, 2011).

[14] Funke J (2001). Dynamic systems as tools for analyzing human judgement. Think. Reason..

[15] Johnson MMS (1990). Age differences in decision making: a process methodology for examining strategic information processing. J. Gerontol..

[16] Orasanu J (2005). Crew collaboration in space: a naturalistic decision-making perspective. Aviat. Space Environ. Med..

[17] Reiskamp, J. & Otto, P. E. in *Heuristics The Foundations of Adaptive Behavior*. (eds Gigerenzer, G., Hertwig, R. & Pachur, T.) Ch. 11 (Oxford University Press, Oxford, England, UK, 2011).

[18] Law J (2014). Relationship between carbon dioxide levels and reported headaches on the International Space Station. J. Occup. Environ. Med..

[19] Cowings, P. S. et al. *Converging Indicators for Assessing Individual Differences in Adaptation to Extreme Environments: Preliminary Report*. NASA/TM-2006-213491. https://ntrs.nasa.gov/archive/nasa/casi.ntrs.nasa.gov/2006005506.pdf (2006). 17547321

[20] Seaton, K. A., Kane, R. L. & Sipes, W. *Cognitive Assessment during Long-duration Space Flight*. NASA/TM*-*2010*-*36584. https://ntrs.nasa.gov/search.jsp?R=20100036584 (2010).

[21] Strangman G, Sipes W, Beven G (2014). Human cognitive performance in spaceflight and analogue environments. Space Environ. Med..

[22] De La Torre GG (2012). Future perspectives on space psychology: Recommendations on psychosocial and neurobehavioural aspects of human spaceflight. Acta Astronaut..

[23] Roalf DR (2014). Neuroimaging predictors of cognitive performance across a standardized neurocognitive battery. Neuropsychology.

[24] Basner M (2015). Development and validation of the Cognition test battery. Aerosp. Med. Hum. Perform..

[25] Curran-Everett D (2000). Multiple comparisons: philosophies and illustrations. Am. J. Physiol. Regul. Integr. Comp. Physiol..

[26] MacNaughton P (2017). The impact of working in a green certified building on cognitive function and health. Bldg. Environ..

[27] Jones, J. M. In U.S., 40% get less than recommended amount of sleep. *Well Being*. http://www.gallup.com/poll/166553/less-recommended-amount-sleep.aspx (2013).

[28] Maddalena R (2015). Effects of ventilation rate per person and per floor area on perceived air quality, sick building syndrome symptoms, and decision‐making. Indoor Air.

[29] Maula H, Hongisto V, Naatula V, Haapakangas A, Koskela H (2016). The effect of low ventilation rate with elevated bioeffluent concentration on work performance, perceived indoor air quality, and health symptoms. Indoor Air.

[30] Guyenet PG, Stornetta RL, Bayliss DA (2010). Central respiratory chemoreception. J. Comp. Neurol..

[31] Guyenet PG, Bayliss DA (2015). Neural control of breathing and CO_2_ homeostasis. Neuron.

[32] Langhorst P, Schulz B, Schulz G, Lambertz M (1983). Reticular formation of the lower brainstem. A common system for cardiorespiratory and somatomotor functions: discharge patterns of neighboring neurons influenced by cardiovascular and respiratory afferents. J. Auton. Nerv. Syst..

[33] Brian JE (1998). Carbon dioxide and the cerebral circulation. Anesthesiology.

[34] Basner M, Mollicone D, Dinges DF (2011). Validity and sensitivity of a brief psychomotor vigilance test (PVT-B) to total and partial sleep deprivation. Acta Astronaut..

[35] Basner M (2017). Effects of −12° head-down tilt with and without elevated levels of CO_2_ on cognitive performance: the SPACECOT study. J. Appl. Physiol..

[36] Maier KL (2017). Protocol for the Reconstructing Consciousness and Cognition (ReCCognition) study. Front. Hum. Neurosci..

[37] Poulin V, Korner-Bitensky N, Dawson DR (2013). Stroke-specific executive function assessment: a literature review of performance-based tools. Aust. Occup. Ther. J..

[38] Satish U, Streufert S, Elsinger PJ (2006). Measuring executive function deficits following head injury: an application of SMS simulation technology. Psychol. Rec..

[39] Krishnamurthy S (2009). Components of critical decision making and ABSITE assessment: toward a more comprehensive evaluation. J. Grad. Med. Educ..

[40] Satish U, Streufert S (2002). Value of a cognitive simulation in medicine: towards optimizing decision making performance of healthcare personnel. Qual. Saf. Health Care.

[41] Satish, U. et al. Novel assessment of psychiatry residents: SMS simulations. *ACGME Bull.* January, 18–23 (2009).

[42] Streufert, S., Pogash, R. M., Piasecki, M. T., Repman, M. A. & Swezey, R. W. *Data Collection via a Quasi-experimental Simulation. III: Factor Structure and Validity*. Report to the U.S. Army Research Institute for the Behavioral and Social Sciences. http://www.dtic.mil/dtic/tr/fulltext/u2/a173913.pdf (1986).

[43] Swezey RW, Streufert S, Satish U, Siem FM (1998). Preliminary development of a computer-based team performance assessment simulation. Int. J. Cogn. Ergon..

[44] Moore TM (2017). Validation of the cognition test battery for spaceflight in a sample of highly educated adults.. Aerosp. Med. Hum. Perform..

[45] Moore Tyler M., Gur Ruben C., Thomas Michael L., Brown Gregory G., Nock Matthew K., Savitt Adam P., Keilp John G., Heeringa Steven, Ursano Robert J., Stein Murray B. (2017). Development, Administration, and Structural Validity of a Brief, Computerized Neurocognitive Battery: Results From the Army Study to Assess Risk and Resilience in Servicemembers. Assessment.

[46] Gur RC (2001). Computerized neurocognitive scanning: II. The profile of schizophrenia. Neuropsychopharmacology.

[47] Gur RC (2001). Computerized neurocognitive scanning: I. Methodology and validation in healthy people. Neuropsychopharmacology.

[48] Glahn DC, Gur RC, Ragland JD, Censits DM, Gur RE (1997). Reliability, performance characteristics, construct validity, and an initial clinical application of a visual object learning test (VOLT). Neuropsychology.

[49] Ragland JD (2002). Working memory for complex figures: an fMRI comparison of letter and fractal n-back tasks. Neuropsychology.

[50] Glahn DC, Cannon TD, Gur RE, Ragland JD, Gur RC (2000). Working memory constrains abstraction in schizophrenia. Biol. Psychiatry.

[51] Benton AL, Varney NR, Hamsher KD (1978). Visuospatial judgment. A clinical test. Arch. Neurol..

[52] Gur RC (2002). A method for obtaining 3-dimensional facial expressions and its standardization for use in neurocognitive studies. J. Neurosci. Methods.

[53] Gur RC (2010). A cognitive neuroscience-based computerized battery for efficient measurement of individual differences: standardization and initial construct validation. J. Neurosci. Methods.

[54] Perfetti B (2009). Differential patterns of cortical activation as a function of fluid reasoning complexity. Hum. Brain Mapp..

[55] Raven J (2000). The Raven’s progressive matrices: change and stability over culture and time. Cogn. Psychol..

[56] Jewett ME, Dijk DJ, Kronauer RE, Dinges DF (1999). Dose–response relationship between sleep duration and human psychomotor vigilance and subjective alertness. Sleep.

[57] Joy S, Fein D, Kaplan E (2003). Decoding digit symbol: speed, memory, and visual scanning. Assessment.

[58] Van Dongen HPA, Maislin G, Mullington JM, Dinges DF (2003). The cumulative cost of additional wakefulness: dose–response effects on neurobehavioral functions and sleep physiology from chronic sleep restriction and total sleep deprivation. Sleep.

[59] Lejuez CW (2002). Evaluation of a behavioral measure of risk taking: the Balloon Analogue Risk Task (BART). J. Exp. Psychol..

[60] Basner M, Dinges DF (2011). Maximizing sensitivity of the psychomotor vigilance test (PVT) to sleep loss. Sleep.

[61] Doran SM, Van Dongen HP, Dinges DF (2001). Sustained attention performance during sleep deprivation: evidence of state instability. Arch. Ital. Biol..

[62] Lim J, Dinges DF (2008). Sleep deprivation and vigilent attention. Ann. N. Y. Acad. Sci..

