# Supplementary material for: Effects of acute exposures to carbon dioxide on decision making and cognition in astronaut-like subjects
Source: NPJ Microgravity. 2019 Jun 19;5:17. doi: 10.1038/s41526-019-0071-6 (PMC6584569; doi:10.1038/s41526-019-0071-6)
Supplement: Supplementary file 2 — Supplementary Information [file 41526_2019_71_MOESM2_ESM.pdf]

Exposure Session 1				Exposure Session 2				Exposure Session 3				Exposure Session 4			
Date	Group	Subj	Dose	Date	Group	Subj	Dose	Date	Group	Subj	Dose	Date	Group	Subj	Dose
26-Apr	1	2	A	2-May	1	2	C	10-May	1	2	B	17-May	1	2	X
26-Apr	1	3	A	2-May	1	3	C	10-May	1	3	B	17-May	1	3	D
26-Apr	1	4	A	2-May	1	4	C	10-May	1	4	B	17-May	1	4	D
26-Apr	1	5	A	2-May	1	5	C	10-May	1	5	B	17-May	1	5	D
26-Apr	1	6	A	2-May	1	6	C	10-May	1	6	B	17-May	1	6	D
26-Apr	1	12	A	2-May	1	12	C	10-May	1	12	B	17-May	1	12	D
28-Apr	2	1	B	5-May	2	1	D	12-May	2	1	A	19-May	2	1	C
29-Apr	2	7	B	5-May	2	7	D	12-May	2	7	A	19-May	2	7	C
29-Apr	2	8	B	5-May	2	8	D	12-May	2	8	A	19-May	2	8	C
28-Apr	2	9	B	5-May	2	9	D	12-May	2	9	A	19-May	2	9	C
28-Apr	2	10	X	5-May	2	10	D	12-May	2	10	A	19-May	2	10	C
28-Apr	2	11	B	5-May	2	11	D	12-May	2	11	A	19-May	2	11	C
31-May	3	14	D	7-Jun	3	14	A	14-Jun	3	14	C	21-Jun	3	14	B
31-May	3	15	D	7-Jun	3	15	A	14-Jun	3	15	C	21-Jun	3	15	B
31-May	3	17	D	7-Jun	3	17	A	14-Jun	3	17	C	21-Jun	3	17	B
28-Jun	3	18	D	7-Jun	3	18	A	14-Jun	3	18	C	21-Jun	3	18	B
2-Jun	4	13	C	9-Jun	4	13	B	16-Jun	4	13	D	23-Jun	4	13	A
5-Jul	4	16	C	9-Jun	4	16	B	16-Jun	4	16	D	23-Jun	4	16	A
2-Jun	4	19	C	9-Jun	4	19	B	16-Jun	4	19	D	30-Jun	4	19	A
2-Jun	4	20	C	9-Jun	4	20	B	16-Jun	4	20	D	23-Jun	4	20	A
2-Jun	4	21	C	9-Jun	4	21	B	16-Jun	4	21	X	23-Jun	4	21	A
2-Jun	4	22	C	9-Jun	4	22	B	16-Jun	4	22	D	23-Jun	4	22	A

Supplementary Table 1. Sequences of Exposures to CO_2 Concentrations. The sequences of exposures is given for each group (1-4) of subjects. Dates and subject identification numbers identified with red font and fill indicate missed sessions, those identified with green font and fill indicate make up sessions. Codes: A = 2500 ppm, B = 1200 ppm, C = 600 ppm, D = 5000 ppm

Supplementary Methods

Subject Criteria

The following criteria were used in the selection of subjects:

- Mixed gender, as close to 50/50 male:female ratio as can be achieved.
- Age 30-55.
- Technical skills demonstrated through professional experience. Advanced degree (e.g. M.S. degree) is desirable, or equivalent years of experience.
- Below are some examples of astronaut selection criteria that will guide subject selection:
    - The requirements for Astronaut Candidates are a bachelor's degree from an accredited institution in engineering, biological science, physical science, or mathematics. Quality of academic preparation is important.
    - Degree must be followed by at least 3 years of related, progressively responsible, professional experience or at least 1,000 hours of pilot-in-command time in jet aircraft.
    - An advanced degree is desirable and may be substituted for experience as follows: master's degree = 1 year of experience,
Doctoral degree = 3 years of experience.
    - Teaching experience, including experience at the K – 12 levels, is considered to be qualifying experience for the Astronaut Candidate position; therefore, educators are encouraged to apply.
    - Additional requirements include the ability to pass the NASA long-duration space flight physical, which includes the following specific requirements: Distant and near visual acuity must be correctable to 20/20 in each eye, and blood pressure not to exceed 140/90 measured in a sitting position.

Subjects were excluded from the study if, during screening examinations subjects were found to:

- Have intracranial pressure of 21mmHg or higher,
- Have any systemic conditions that could affect the visual system,
- Have >70% internal carotid artery occlusion
- Require any medication that may interfere with the interpretation of the results
- Have a recent sub-standard nutritional status
- Have a history of thyroid dysfunction, renal stones, mental illness, or smoking within six months prior to the start of the study
- Have a family history of thrombosis
- Have failed a criminal background check
- Have a fasting plasma glucose level less than the American Diabetes Association diagnostic criterion for impaired glucose tolerance (equal to or greater than 100 mg/dl).
- Have a low ferritin level
- Have ingested anabolic steroids within 6 months.
- All subjects are required to pass a modified Air Force Class III Physical examination, or equivalent, within 1 year prior to start of study, and each must be capable of giving informed consent. The tests to be performed include vital signs, 12-lead Electrocardiogram, vision and audiometry, urine analysis, full physical exam, electrolyte

panel, complete blood count with differential, test of liver function, drug screen, lipid panel, chest X-ray, human immunodeficiency virus/hepatitis screen, and high-sensitivity C-reactive protein.
